# Supplementary material for: Women’s decisions regarding family planning use and its determinants in Ethiopia: A systematic review and meta-analysis protocol
Source: PLoS One. 2022 Oct 13;17(10):e0276128. doi: 10.1371/journal.pone.0276128 (PMC9560486; doi:10.1371/journal.pone.0276128)
Supplement: S3 File — (DOCX) [file pone.0276128.s003.docx]

**Additional file 3: Databases search terms**

**PubMed**

((((((((((((((((((((((((((((((((Personal Autonomy[MeSH Terms]) OR (Personal Autonomy[Title/Abstract])) OR (Autonomy, Personal[Title/Abstract])) OR (Self Determination[Title/Abstract])) OR (Free Will[Title/Abstract])) OR (Decision Making[MeSH Terms])) OR (Decision Making[Title/Abstract])) OR (Empowerment[MeSH Terms])) OR (Empowerment[Title/Abstract])) OR (Family Planning Services[MeSH Terms])) OR (Family Planning Services[Title/Abstract])) OR (Family Planning Service[Title/Abstract])) OR (Planning Service, Family[Title/Abstract])) OR (Planning Services, Family[Title/Abstract])) OR (Service, Family Planning[Title/Abstract])) OR (Services, Family Planning[Title/Abstract])) OR (Family Planning[Title/Abstract])) OR (Pregnancy, Planned[Title/Abstract])) OR (Planned Pregnancies[Title/Abstract])) OR (Pregnancies, Planned[Title/Abstract])) OR (Planned Pregnancy[Title/Abstract])) OR (Family Planning Programs[Title/Abstract])) OR (Family Planning Program[Title/Abstract])) OR (Program, Family Planning[Title/Abstract])) OR (Programs, Family Planning[Title/Abstract])) OR (Contraceptive Agents, Female[MeSH Terms])) OR (Contraceptive Agents, Female[Title/Abstract])) OR (Agents, Female Contraceptive[Title/Abstract])) OR (Female Contraceptive Agents[Title/Abstract])) OR (Contraceptives, Female[Title/Abstract])) OR (Female Contraceptives[Title/Abstract])) AND (((Ethiopia[MeSH Terms]) OR (Ethiopia[Title/Abstract])) OR (Federal Democratic Republic of Ethiopia[Title/Abstract]))) AND ((((((((Women[MeSH Terms]) OR (Women[Title/Abstract])) OR (Girls[Title/Abstract])) OR (Girl[Title/Abstract])) OR (Woman[Title/Abstract])) OR (Women's Groups[Title/Abstract])) OR (Women Groups[Title/Abstract])) OR (Women's Group[Title/Abstract]))

**Google scholar**

allintitle: "Women's decision in family planning use" AND Ethiopia

allintitle: "family planning use" AND "Women's decision"

allintitle: "family planning use" AND "Women's decision" Ethiopia

allintitle: "family planning use" AND Ethiopia

allintitle: "contraceptive use" AND Ethiopia

**African Journals online**

“Women’s decisions regarding family planning use in Ethiopia”

**CINAHL**

(MM "Decision Making+") OR (MM "Family Planning+") OR (MM "Birth Intervals")

(MM "Ethiopia")

(MM "Women+") OR (MM "Female")

(MM "Social Determinants of Health") OR (MM "Risk Factors+")
