# Supplementary material for: Women’s decisions regarding family planning use and its determinants in Ethiopia: A systematic review and meta-analysis protocol
Source: PLoS One. 2022 Oct 13;17(10):e0276128. doi: 10.1371/journal.pone.0276128 (PMC9560486; doi:10.1371/journal.pone.0276128)
Supplement: Attachment — Submitted filename: Respons to reviewers.docx [file pone.0276128.s005.docx]

**Dear, editor, and reviewer**

**Editor Comments**

≠ Authors did not provide details of the specific inconsistencies contained in the published findings related to women’s decision-making about family planning and the use of contraceptives in Ethiopia.

- Addressed line 107-113

≠ Line 75-77: Lack of details regarding how having greater decision-making power by women will result in the stated benefits.

- Addressed line 73-76

≠ Line 77: Lack of details regarding the peculiar factors (social, cultural or economic) that predispose women in Africa to deprivation of reproductive rights? How does this affect their decision-making power during family planning discussions or choices?

- Addressed line 77-84

≠Line 78: Why is that? What factors underline this? Is this cultural or economic? What factors underline this? Is this linked to the lack of decision-making power for women concerning family planning?

- Addressed line 77-84

≠Line 89-92: How? Provide details of how these factors have affected women decision-making power regarding family planning.

- **Addressed line 99-102**

≠ Line 97-99: What were the major findings of these studies? What are the inconsistencies in the data / findings generated from these studies? This is a crucial gap in the introduction section which will provide a solid justification for conducting your SR/MA.

- **Addressed line 109-113**

≠ Line 110: Lack of clarity regarding the specific component / aspect of women's decision-making that will be focused of the SR/MA.

- **Addressed line 113-117**

**Reviewer comments**

Reviewer #1: The manuscript is poorly presented and difficult to read and understand. Scientific language is lacking for terms used, research questions and objectives are poorly explained, the methodology is difficult to understand and the discussion is lacking depth and coherence.

- Language edited. Revised Manuscript with Track Changes and the clean manuscript was uploaded on the online submission.
