# Supplementary material for: Women’s decisions regarding family planning use and its determinants in Ethiopia: A systematic review and meta-analysis protocol
Source: PLoS One. 2022 Oct 13;17(10):e0276128. doi: 10.1371/journal.pone.0276128 (PMC9560486; doi:10.1371/journal.pone.0276128)
Supplement: Attachment — Submitted filename: Respons to reviewers.docx [file pone.0276128.s006.docx]

**Dear, editor, and reviewer**

**Editor Comments**

≠ Please update your Cover Letter to indicate whether you wish to have the manuscript considered as a Study Protocol, and confirm that neither recruitment nor data collection have been completed.

- Addressed and uploaded on the online submission “Cover letter” file

Additional Editor Comments

≠ 1. Kudos to the authors for revising the manuscript for grammar, syntax and clarity. However, few minor edits for grammar remain. Please, check the manuscript to identify the areas requiring minor revision for grammar

- Checked and uploaded on the “revised manuscript with track changes” file

≠ 2. The response provided by the authors did not address my previous comment regarding ≠ Line 75-77 in the previous manuscript: Lack of details regarding how having greater decision-making power by women will result in the stated benefits. The revision done in line 73-76 of the revised manuscript did not address the comments. Please, revise this section accordingly.
Addressed line 73-76.

- Addressed on line 73-78

≠3. The response provided by the authors in line 77-84 of the revised manuscript did not address my previous comment regarding ≠ Line 77: Lack of details regarding the peculiar factors (social, cultural or economic) that predispose women in Africa to deprivation of reproductive rights? How does this affect their decision-making power during family planning discussions or choices? Please, revise the manuscript accordingly.

- Addressed on line 79-88

**Reviewer comments**

#1: [NOTE: If reviewer comments were submitted as an attachment file, they will be attached to this email and accessible via the submission site. Please log into your account, locate the manuscript record, and check for the action link "View Attachments". If this link does not appear, there are no attachment files.]

- The link "View Attachments" does not appear

#2: While revising your submission, please upload your figure files to the Preflight Analysis and Conversion Engine (PACE) digital diagnostic tool, <https://pacev2.apexcovantage.com/>. PACE helps ensure that figures meet PLOS requirements. To use PACE, you must first register as a user. Registration is free. Then, login and navigate to the UPLOAD tab, where you will find detailed instructions on how to use the tool. If you encounter any issues or have any questions when using PACE, please email PLOS at [figures@plos.org](mailto:figures@plos.org). Please note that Supporting Information files do not need this step.

- As this is a protocol, there is no figure. Only supporting information’s are available and uploaded on the online submission.
