# Supplementary material for: Women’s decisions regarding family planning use and its determinants in Ethiopia: A systematic review and meta-analysis protocol
Source: PLoS One. 2022 Oct 13;17(10):e0276128. doi: 10.1371/journal.pone.0276128 (PMC9560486; doi:10.1371/journal.pone.0276128)
Supplement: Attachment — Submitted filename: Respons to reviewers.docx [file pone.0276128.s007.docx]

**Dear, editor, and reviewer**

**Editor Comments**

The manuscript is still filled with grammatical and syntax errors in several areas, and these errors must be addressed with professional editing. A few of the examples of errors are stated below:
Line 75: “increase to make choices”

- Corrected line 75-76

Line 77: “increase awareness of women's subordination”

- Corrected line 77

Line 81: “such as the low educational status of women and partners;;”

- Corrected line 81

Line 83” “antennal care visits;;”

- Corrected line 83

Line 85: “such us attitude of family planning methods;;”

- Corrected line 85

Line 91-92” “In developing countries, contraceptive use was low (40%), and the unmet need for family planning was 225 million people [20].”

- Corrected line 90-91

Line 93-94: “In Ethiopia, contraceptive use was low (41.4%), and there was a high unmet need for family planning use (22%).”

- Corrected line 92-93

Line 98: “Low consumption‒consumption”

- Corrected line 97

Line 104: “and reproductive health services”

- Corrected line 102-103

Line 113-118: “Moreover, there are studies conducted in different parts of the country on women's decision-making in family planning [8, 25, 31-35]; however, there are inconsistent findings, with a prevalence of women’s decisions regarding family planning use ranging from 35.9% to 98%, and different factors affecting women’s decisions regarding family planning use were reported among the studies, including residence, knowledge, attitude, educational status, occupational status, age, income, husband influence, and number of living children.”

- Corrected line 111-117

Line 180-183: “The primary outcome of the study was the pooled prevalence of women’s decision making regarding family planning use. Women’s decision making regarding family planning was considered whether women have decision-making power regarding delayed marriage”

- Corrected line 174-176

Line 185-186: “The secondary outcome of the study was to identify determinates of women’s decision making regarding family planning use”

- Corrected line 179-180

Line 192-193: “online databases and gray literature will be used to search studies.”

- Corrected line 186

Line 194-195: “Independent search strategies will be performed by (EW and SB).”

- Corrected line 188
